# Aspirin Compared to Other Thromboprophylactic Agents in Patients Following Total Hip Arthroplasty: A Literature Review

**DOI:** 10.7759/cureus.65645

**Published:** 2024-07-29

**Authors:** Joseph Hanna, Rahel Rashid, Mark Hanna, Beshoy Effat Elkomos, Guirgis Ebeidallah

**Affiliations:** 1 Trauma and Orthopaedics, Wirral University Teaching Hospital NHS Foundation Trust, Wirral, GBR; 2 General and Colorectal Surgery, Arrowe Park Hospital, Wirral, GBR; 3 Vascular Surgery, Countess of Chester Hospital, Chester, GBR; 4 General and Emergency Surgery, Royal United Hospital, Bath, GBR; 5 Emergency Medicine, Salford Royal NHS Foundation Trust, Salford, GBR

**Keywords:** total hip arthroplasty (tha), pulmonary embolism (pe), aspirin, thromboprophylaxis, venous thromboembolism (vte)

## Abstract

Total hip arthroplasty (THA) is a common but major surgery performed in the United Kingdom and around the globe. THA is associated with several postoperative complications, with one of the most common being venous thromboembolism (VTE) in the form of deep venous thrombosis (DVT) or pulmonary embolism (PE). VTE following orthopaedic surgery can have major consequences in terms of patient morbidity and may even cause mortality. It carries a significant cost to the health service, and thromboprophylactic agents are used to decrease the risk. Several different options are available for chemical thromboprophylaxis, including aspirin, low-molecular-weight heparin (LMWH), direct oral anticoagulants (DOACs), and warfarin. This study aims to review the literature to determine if aspirin is less superior to the other available chemical thromboprophylaxis in postoperative patients following THA. The primary outcome assessed in this review is rates of symptomatic 90-day VTE in the form of PE or DVT. A literature review was conducted using PubMed, Scopus, and Google Scholar using the following terms: ‘Aspirin AND (low molecular weight heparin OR LMWH OR Enoxaparin OR Apixaban OR DOAC OR direct oral anticoagulant OR warfarin) AND (orthopaedic OR orthopedic) AND (Total hip replacement OR THR OR THA OR total hip arthroplasty) AND (‘venous thromboembolism’ OR VTE).’ Aspirin appears to have promising results as thromboprophylaxis in cases of THA. However, it is still up for debate as to whether it is non-inferior to other forms of thromboprophylaxis.

## Introduction and background

Total hip arthroplasty (THA) is a common and highly effective surgical intervention performed in the United Kingdom, the United States, and around the world [1,2]. Although it is most commonly performed for osteodegenerative conditions such as osteoarthritis, it has also been recently utilised in certain trauma situations [3,4].

Like any form of surgical intervention, THA is not without complications. One of the most commonly encountered issues is venous thromboembolism (VTE), particularly in the form of deep venous thrombosis (DVT) and/or pulmonary embolism (PE), with an incidence of approximately 0.6-1.5% [5]. VTE following orthopaedic surgery can have major consequences in terms of patient morbidity and may even cause mortality [6,7]. It carries a significant cost to the health service, costing about $15,000 to $30,000 per episode [8,9].

VTE encompasses both DVT and PE. DVT typically manifests as pain, erythema, warmth, or swelling of the affected limb, most commonly in the lower limbs, although it can also occur in the upper limbs [10].

Virchow’s triad has been found to be associated with VTE and includes endothelial injury, venous stasis, and hypercoagulable state. Two of these factors have to be met for VTE to develop [11]. Frequently, in THA, the patient is predisposed to develop all three components of the triad. Prolonged immobilisation, inability to bear weight, tourniquet use, intraoperative dissection and manipulation of tissues, and the body’s hormonal and neuronal response to the trauma that an operation will lead to the development of all three and subsequent VTE [12-14].

The rate of DVT development following THA without providing pharmacologic prophylaxis has been reported to be as high as 50%. Therefore, the significance of pharmacologic and multimodal thromboprophylaxis is evident [15]. The question remains as to which form of pharmacologic thromboprophylaxis to prescribe to patients.

There has been a recent trend in using aspirin for VTE prophylaxis. The growing utilisation of aspirin as thromboprophylaxis is supported by mounting evidence of its safety and efficacy. Matharu et al. conducted a meta-analysis of 13 randomised controlled trials (RCTs) evaluating aspirin for thromboprophylaxis, revealing a relative risk of 1.12 (95% confidence interval (CI) = 0.78-1.62) compared to other pharmacologic regimens [16].

## Review

Methodology

A literature review was conducted using PubMed, Scopus, and Google Scholar using the following terms: ‘Aspirin AND (low molecular weight heparin OR LMWH OR Enoxaparin OR Apixaban OR DOAC OR direct oral anticoagulant OR warfarin) AND (orthopaedic OR orthopedic) AND (Total hip replacement OR THR OR THA OR total hip arthroplasty) AND (‘venous thromboembolism’ OR VTE).’ Results were restricted to the past five years to review the most recent advances regarding this topic, duplicates were removed, and secondary research was excluded to obtain the most recent literature in the field. The remainder of the results were vetted by reading the articles. Finally, six articles were included in this review. Jamovi software (Version 2.5.7.0) was used for statistical analysis.

Results

Five of the six selected studies were retrospective comparative cohort studies while the CRISTAL study was the only prospective RCT. All studies were published between 2020 and 2024 and examined between two to eleven years worth of data. A summary of the study characteristics can be viewed in Table 1 in a PICO format.

**Table 1:** PICO of the six primary studies reviewed. PICO = Population, Intervention, Comparison, Outcome; THA = total hip arthroplasty; DOAC = direct oral anticoagulant; LOS = length of stay; VTE = venous thromboembolism; LMWH = low-molecular-weight heparin

	Population (patients undergoing THA)	Intervention	Comparison	Outcomes assessed
Matharu et. al. [17]	Total = 104,040 patients available. Matched confounding variables 28,049 in both groups: Aspirin vs. direct thrombin inhibitors; 19,021 in both groups: aspirin vs. factor Xa inhibitors	Aspirin	Non-aspirin (DOACs and factor Xa inhibitors)	90-day VTE, LOS, and adverse events
Habibi et. al. [18]	Total n = 1,220; aspirin n = 214; non-aspirin = 1,006 (however, this was then matched into cohorts of 214 each)	Aspirin	Non-aspirin (enoxaparin)	Hospital LOS, discharge disposition, discharge VTE prophylaxis, 90-day diagnosis of VTE, 90-day emergency department visit, and 90-day readmission
Sidhu et al. [19]	Total n = 1,867; aspirin alone n = 365 (20%); non-aspirin n = 1,502 (however, of those, 482 (26%) used LMWH with aspirin concurrently or sequentially)	Aspirin	Non-aspirin (LMWH, LMWH and aspirin, DOAC)	Primary: symptomatic 90-day VTE. Secondary: major bleeding, joint-related reoperation, and mortality within 90 days
Moore et al. [20]	Total n = 78,907; aspirin n = 36,346; non-aspirin n= 42, 561 (dabigatran n = 13,065, rivaroxaban n = 11,790, enoxaparin n = 11,380, and warfarin n = 6,326)	Aspirin	Non-aspirin (dabigatran, rivaroxaban, enoxaparin, and warfarin)	90-day postoperative VTE and transfusion
Singh et al. [21]	Total n = 35,142; aspirin n = 14,731; non-aspirin n = 20,411	Aspirin	Non-aspirin (DOAC, LMWH, warfarin)	Primary: 90-day VTE. Secondary: 90-day deep infection, readmission, bleeding event, wound complication, and mortality
Sidhu et al. (CRISTAL study) [22]	Total n = 9203 patients; aspirin n= 5,146; enoxaparin n = 7,238	Aspirin	Non-aspirin (enoxaparin)	Primary: symptomatic 90-day VTE. Secondary: death and major bleeding within 90 days (six secondary outcomes overall)

Further characteristics of the included studies are characterised in Table 2.

**Table 2:** Study characteristics.

Authors	Date published	Journal	Study design	Statistical tests used
Matharu et. al. [17]	May 2020	The Journal of Arthroplasty	A retrospective, comparative, observational study conducted over 9 years	Standardised mean difference
Habibi et al. [18]	January 2024	European Journal of Orthopaedic Surgery & Traumatology	A retrospective, non-inferiority study conducted over 11 years	Categorical - Fisher’s exact tests and chi-square tests. Continuous - two-tailed t-test
Sidhu et al. [19]	August 2023	BMC Musculoskeletal Disorders	A multicentre, retrospective, comparative, observational study conducted over 2 years	Logistic regression, Student’s t-test, and Fisher’s exact test
Moore et al. [20]	November 2023	Journal of Orthopaedics	A retrospective, comparative, observational study conducted over 6 years	Student’s t-test and chi-square test
Singh et al. [21]	February 2023	The Journal of Arthroplasty	A retrospective, non-inferiority cohort study conducted over 10 years	Non-inferiority
Sidhu et al. (CRISTAL study) [22]	August 2022	JAMA	A cluster-randomized, crossover, registry-nested trial	Non-inferiority at a margin of 1%

Matharu et al. [17] conducted the first study reviewed in our cohort, published in May 2020. This retrospective, observational study utilised the largest available sample, examining nine years of data from the world’s largest mandatory registry for joint replacement. The primary outcome was to determine the 90-day VTE occurrence rate, while the secondary outcomes included length of stay (LOS) and adverse events. The study included 104,040 patients. After propensity matching for confounding variables, the patients were divided into two groups. The first group, consisting of 28,049 patients each, compared the outcomes of aspirin with direct oral anticoagulants (DOACs). The second group, also comprising 19,021 patients each, compared aspirin with factor Xa inhibitors. The standardised mean difference was primarily used for statistical analysis. In the first group, the VTE rate was reported as 177/28,049 (0.63%) for aspirin users, compared to 123/28,049 (0.44%) for those taking DOACs, with a p-value of 0.002. In the second group, those on aspirin had a VTE rate of 112/19,021 (0.59%), compared to 70/19,021 (0.37%) for those using factor Xa inhibitors, with a p-value of 0.003. The study concluded in favour of non-aspirin VTE prophylaxis [17].

Habibi et. al. published a non-inferiority study, retrospectively examining 11 years of patient data. Using propensity score matching, outcomes for patients prescribed aspirin (n = 214) were compared to those for patients prescribed non-aspirin VTE prophylaxis (n = 1,006). No significant risk-adjusted differences were observed in the incidence of VTE (0.5 vs. 0.5%), with a p-value of 1.000, between patients prescribed aspirin and those receiving non-aspirin thromboprophylaxis, respectively. The same was observed for 90-day readmissions (10.4% and 12.3%; p = 0.646). Patients on non-aspirin prophylaxis exhibited increased rates of non-home discharge (73.9% vs. 58.5%; p < 0.001) and extended length of hospital stay (143.5 hours vs. 124.9 hours; p = 0.005) [18].

Sidhu et al. conducted a multicentre observational study focusing on VTE rates among patients receiving aspirin and other forms of VTE prophylaxis. The study also investigated secondary outcomes such as major bleeding events, joint-related reoperations, and mortality within a 90-day timeframe. The study included 1,876 patients categorised into groups receiving aspirin alone (20%), LMWH alone (41%), a combination of the two (26%), and DOACs (9%). They reported a 90-day VTE rate of 2.7%, with the lowest incidence observed in the aspirin group (1.6%) compared to 3.6% for LMWH alone, 2.3% for LMWH combined with aspirin, and 2.4% for DOACs [19].

Moore et al. analyzed a national database to assess 90-day VTE rates and the need for blood transfusion. Notably, between 2016 and 2021, they observed the greatest increase in aspirin use and the largest decline in dabigatran use. They reported the frequency of use in their cohort as follows: aspirin (36,346; 46%), dabigatran (13,065; 16.5%), rivaroxaban (11,790; 15%), and warfarin (6,326; 8%). The reported rates of 90-day VTE were 0.4%, 3.9%, 2.5%, 1.2%, and 2.2%, respectively (p < 0.0001). Regarding blood transfusion within 90 days postoperatively, the rates were 1.1%, 1.4%, 1.4%, 1.9%, and 1.6%, respectively (p < 0.0001) [20].

Singh et al. conducted another retrospective study, spanning 10 years (from 2009 to 2019). Their primary aim was to compare the efficacy of aspirin to LMWH, factor Xa inhibitors, and warfarin in high-risk VTE cases undergoing THA. They had a cohort of 35,142 THA cases. A validated VTE risk assessment score was utilised to measure risk, and the 90-day incidence of VTE was assessed with propensity score-weighted logistic regression. VTE occurred in 0.5% of those on aspirin compared to 0.4% (DOAC), 0.9% (LMWH), and 0.9% (warfarin). Aspirin was not inferior to LMWH (odds ratio (OR) = 0.59) nor warfarin (OR = 0.69). They found insufficient evidence to support the non-inferiority of aspirin compared to potent anticoagulants in patients undergoing higher-risk THA [21].

The CRISTAL study by Sidhu et al. is the only randomised clinical trial included in this review. This was a cluster randomisation (into aspirin 100 mg and LMWH 40 mg) across various hospitals performing THA to determine the non-inferiority of aspirin compared to LMWH in VTE prevention. The reported 90-day VTE rate was 3.45% in those who were prescribed aspirin and 1.82% in the LMWH group (estimated difference = 1.97%; 95% CI = 0.54%-3.41%; p = 0.007) [22]. Other statistically significant results are shown in Table 3.

**Table 3:** Relevant primary and secondary outcomes. VTE = venous thromboembolism; LOS = length of stay; DOAC = direct oral anticoagulants; LMWH = low-molecular-weight heparin

	Rates of VTE (primary outcome), N (%)	Secondary outcomes (statistically significant results)	Conclusions
Matharu et al. [17]	Aspirin = 177/28,049 (0.63); direct thrombin inhibitors = 123/28,049 (0.44); p-value = 0.002. Aspirin = 112/19,021 (0.59); factor Xa inhibitor = 70/19,021 (0.37); p-value = 0.003	Length of stay: Aspirin vs. direct thrombin inhibitor = −0.37 days (−0.43 to −0.31); p < =.001. Aspirin vs. factor Xa inhibitor = −0.80 days (−0.87 to −0.74); p < 0.001	Favours non-aspirin
Habibi et al. [18]	Aspirin = 1/214 (0.5); enoxaparin = 2/214 (0.9); p-value = 1.000	LOS mean hours (range): Aspirin = 124.9 (32.0–317.0); enoxaparin = 140.8 (49.0–377.0); p-value = 0.024. Non-home discharge, N (%): Aspirin = 124 (58.8); enoxaparin = 160 (75.8)	No statistically significant difference
Sidhu et al. [19]	Aspirin = 6/363 (1.6); LMWH = 27/758 (3.6); LMWH/Aspirin = 11/478 (2.3); DOAC = 4/170 (2.4); p-value = 0.6	No statistically significant secondary outcomes	No statistically significant difference
Moore et al. [20]	Aspirin = 153/36,346 (0.4); dabigatran = 512/13,065 (3.9); rivaroxaban = 290/11,790 (2.5); enoxaparin = 132 /11,380 (1.2); warfarin = 138 /6326 (2.2); p-value < 0.0001	Transfusion, N (%): Aspirin = 394 /36,346 (1.1); dabigatran = 182 /13,065 (1.4); rivaroxaban = 170 /11,790 (1.4); enoxaparin = 215 /11,380 (1.9); warfarin - 103/6,326 (1.6); p-value < 0.0001	Favours aspirin
Singh et al. [21]	Total n = 35,142; Aspirin = 78/14,731 (0.5); DOAC = 5/1,167 (0.4); LMWH = 84/ 9,277 (0.9); p-value = 0.013; warfarin – 92/9,967 (0.9); p-value = 0.016	Deep infection: Aspirin = 64/14,731 (0.4); warfarin = 85/9,967 (0.9); p-value = 0.001. Readmission: Aspirin = 585 /14,731 (4.0); warfarin = 686 /9,967 (6.9); p-value < 0.001. Bleeding event: Aspirin = 32 /14,731 (0.2); warfarin = 66 /9,967 (0.7); P-value < 0.001	Favours aspirin
Sidhu et al. (CRISTAL study) [22]	Aspirin = 187/5416 (3.45); enoxaparin = 69/3787 (1.8); p-value = 0.007	No statistically significant secondary outcomes	Favours non-aspirin

**Figure 1:**
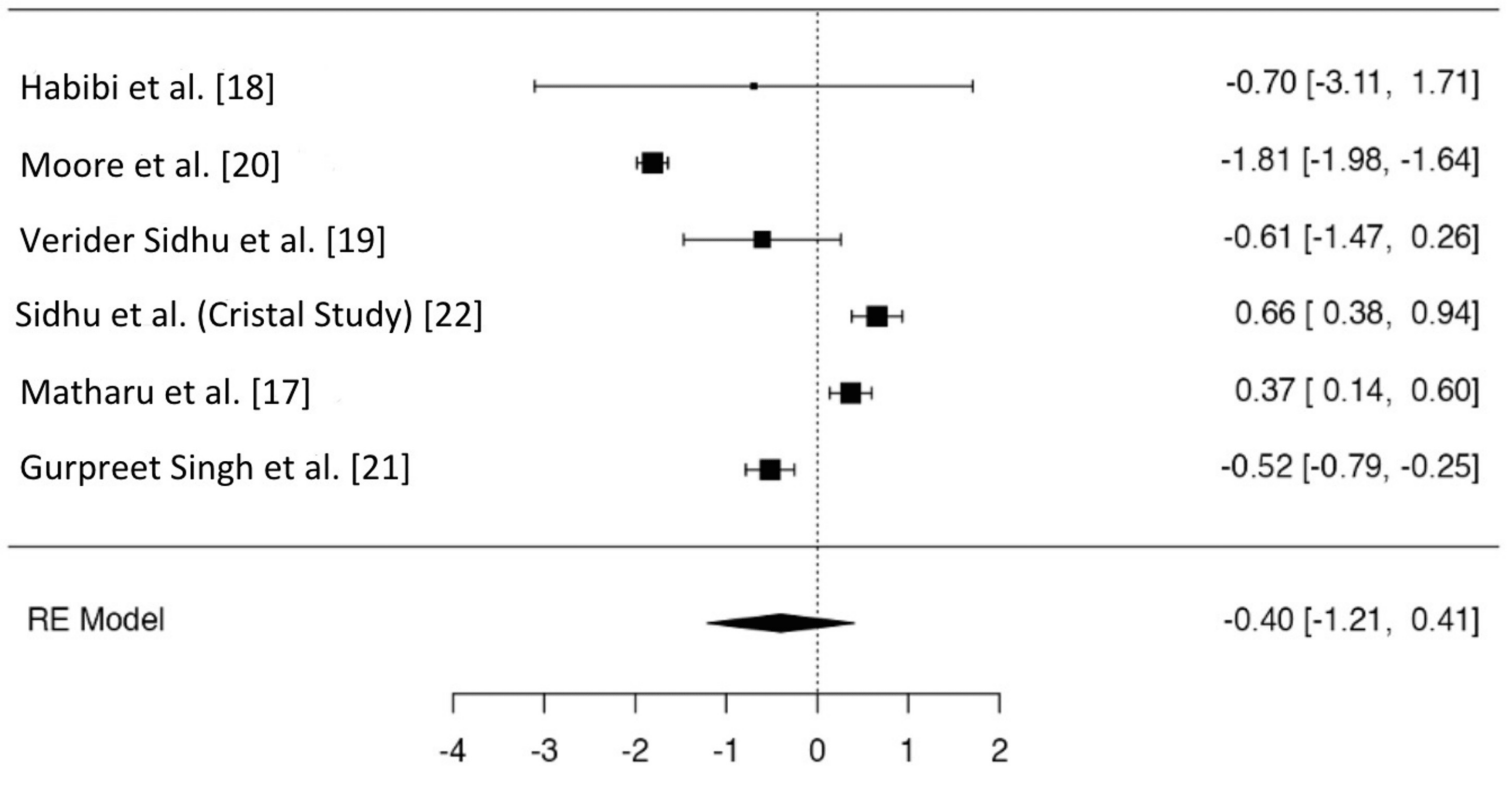
Forest plot of the six included studies using log odds ratio. RE = random effects

A forest plot using the OR of VTE rates from all six studies is shown in Figure 1.

A total of six studies were included in the analysis. The observed log ORs ranged from -1.8103 to 0.6560, with the majority of estimates being negative (67%). The estimated average log OR based on the random-effects model was \hat{\mu} = -0.4011 (95% CI = -1.2080 to 0.4058). Therefore, the average outcome did not differ significantly from zero (z = -0.9744, p = 0.3299).

Discussion

A myriad of guidelines exist worldwide regarding the optimal thromboprophylaxis regime for patients who undergo hip arthroplasty operations. This has resulted in significant ambiguity and differing opinions. The National Institute of Health and Care Excellence in the United Kingdom does not recommend using aspirin as thromboprophylaxis, while the American Academy of Orthopaedic Surgeons advocates for its use [23]. Historically, patients who underwent THA without the cover of thromboprophylaxis have had VTE rates of up to 50% [15]. VTE rates have been much lower recently owing to a multimodal approach to thromboprophylaxis, and, on the other hand, bleeding rates (from the different types of chemical thromboprophylaxis) may be more concerning to some. Hence, the scientific discussion has become more focused towards optimising VTE thromboprophylaxis for the sake of both risks. We set out to review the recent literature (within the last five years) surrounding this debate and for updates on this contention.

The six articles included in this review were of varying quality and strength, but they all measured rates of VTE as their primary outcome of interest. Five of them were retrospective, observational studies and only one was an RCT.

Outcomes of Venous Thromboembolism

The outcome of most interest in this review was symptomatic 90-day VTE. All articles assessed this as their primary outcome. There were, however, variations between the results, leading to differing conclusions.

Two of the five retrospective observational studies found no significant difference in 90-day VTE rates [17,18]. Although not statistically significant, Sidhu et al. did report the aspirin cohort having the lowest rate of 90-day VTE (1.6%) compared to LMWH (3.6%), a combination of LMWH and aspirin (2.3%), and DOACs (2.4%). The authors did comment that this comparatively lower rate of VTE was likely due to selective use in lower-risk patients. These studies had the lowest sample sizes of the six selected studies with only 214 and 365 patients in the aspirin cohorts, respectively.

Both Moore et al. and Singh et al. published studies showing aspirin reduced the rates of VTE post-THA [20,21]. These studies used registry data and were similar in method and boasted large sample sizes. They were both conducted in the United States where aspirin thromboprophylaxis is most commonly used. Moore et al. reported a rate of 0.4% VTE in those using aspirin which was statistically significantly lower than all other chemoprophylactic options which had rates ranging from 1.2% to 3.9% (p < 0.0001). Both these studies gathered data from the United States where aspirin tends to be used in patients at low risk of VTE. This likely contributed to the lower rates of VTE in the aspirin groups. Moore et al. did not perform any propensity matching statistical analysis to try to mitigate this bias. When Singh et al. performed propensity matching, the event rate (VTE) was too rare to evaluate higher-risk THA; therefore, results were no longer statistically significant.

The remaining two studies demonstrated that aspirin increased the risk of VTE when compared to other thromboprophylaxis. These studies had stronger evidence as the CRISTAL study is the only prospective RCT, and although Matharu et al. is another retrospective study, they had the largest original data available and were able to use propensity matching to equalise confounders while still maintaining a large sample size, unlike the previously discussed studies. Matharu et al. presented a VTE rate of 0.59% and 0.63% compared to 0.37% and 0.44% when comparing aspirin to direct thrombin inhibitors and factor Xa inhibitors, respectively [17,22].

Secondary Outcomes

The main secondary outcome of interest was bleeding risk. This was assessed in several different ways in the studies. Some assessed major bleeding events, others assessed any bleeding event, while Moore et al. assessed transfusion rates.

Of all six studies, only two had a statistically significant difference in bleeding risk. Singh et al. demonstrated an increased rate of bleeding events when using warfarin compared to aspirin (0.7% vs. 0.2%; p < 0.001); there was no statistical difference when comparing aspirin to DOACs or LMWH [20]. Moore et al. showed that patients taking aspirin were less likely to require transfusions compared to all other types of chemo-thromboprophylaxis (p < 0.0001) [19]. All other studies including the CRISTAL RTC and the propensity-matched retrospective study by Matharu et al. showed no statistical difference in bleeding risk.

When assessing LOS, Matharu et al. demonstrated an increase in LOS in patients receiving aspirin compared to direct thrombin inhibitors (-0.43 to -0.31; p < 0.001) and factor Xa inhibitors (-0.87 to -0.74; p < 0.001) [17]. On the other hand, despite the small sample size, Habibi et al. did show a statistically significant reduction in LOS and non-home discharge in patients taking aspirin compared to enoxaparin (124.9 to 160 hours; p = 0.024) [18]. Although this was statistically significant, as the difference in LOS is roughly just one day extra, the impact of this likely limited.

This is a limited literature review of recent published articles. We have limited this review to the previous five years only. This can introduce significant sampling bias and reduce the number of articles included to only six.

## Conclusions

While evidence for the non-inferiority of aspirin has not been conclusive, a significant body of evidence points to its successful use in low-risk patients undergoing THA. It remains to be seen whether this can be successfully replicated across all patients undergoing THA. Further RCTs examining the non-inferiority of aspirin vs. non-aspirin chemoprophylaxis are warranted, along with further cost-analysis studies.

## References

[1] Ferguson RJ, Palmer AJ, Taylor A, Porter ML, Malchau H, Glyn-Jones S (2018). Hip replacement. Lancet.

[2] Learmonth ID, Young C, Rorabeck C (2007). The operation of the century: total hip replacement. Lancet.

[3] Luo S, Qin W, Yu L, Luo R, Liang W (2023). Total hip arthroplasty versus hemiarthroplasty in the treatment of active elderly patients over 75 years with displaced femoral neck fractures: a retrospective study. BMC Musculoskelet Disord.

[4] Nho SJ, Kymes SM, Callaghan JJ, Felson DT (2013). The burden of hip osteoarthritis in the United States: epidemiologic and economic considerations. J Am Acad Orthop Surg.

[5] Santana DC, Emara AK, Orr MN (2020). An update on venous thromboembolism rates and prophylaxis in hip and knee arthroplasty in 2020. Medicina (Kaunas).

[6] Abe K, Kuklina EV, Hooper WC, Callaghan WM (2019). Venous thromboembolism as a cause of severe maternal morbidity and mortality in the United States. Semin Perinatol.

[7] Bhandari M, Einhorn TA, Guyatt G (2019). Total hip arthroplasty or hemiarthroplasty for hip fracture. N Engl J Med.

[8] Grosse SD, Nelson RE, Nyarko KA, Richardson LC, Raskob GE (2016). The economic burden of incident venous thromboembolism in the United States: a review of estimated attributable healthcare costs. Thromb Res.

[9] Shahi A, Chen AF, Tan TL, Maltenfort MG, Kucukdurmaz F, Parvizi J (2017). The incidence and economic burden of in-hospital venous thromboembolism in the United States. J Arthroplasty.

[10] Hirsh J, Hull RD, Raskob GE (1986). Clinical features and diagnosis of venous thrombosis. J Am Coll Cardiol.

[11] Myers D Jr, Farris D, Hawley A (2002). Selectins influence thrombosis in a mouse model of experimental deep venous thrombosis. J Surg Res.

[12] Frank B, Maher Z, Hazelton JP (2017). Venous thromboembolism after major venous injuries: competing priorities. J Trauma Acute Care Surg.

[13] Roth-Isigkeit A, Borstel TV, Seyfarth M, Schmucker P (1999). Perioperative serum levels of tumour-necrosis-factor alpha (TNF-alpha), IL-1 beta, IL-6, IL-10 and soluble IL-2 receptor in patients undergoing cardiac surgery with cardiopulmonary bypass without and with correction for haemodilution. Clin Exp Immunol.

[14] Dahl OE, Harenberg J, Wexels F, Preissner KT (2015). Arterial and venous thrombosis following trauma and major orthopedic surgery: molecular mechanisms and strategies for intervention. Semin Thromb Hemost.

[15] Morris GK, Henry AP, Preston BJ (1974). Prevention of deep-vein thrombosis by low-dose heparin in patients undergoing total hip replacement. Lancet.

[16] Matharu GS, Kunutsor SK, Judge A, Blom AW, Whitehouse MR (2020). Clinical effectiveness and safety of aspirin for venous thromboembolism prophylaxis after total hip and knee replacement: a systematic review and meta-analysis of randomized clinical trials. JAMA Intern Med.

[17] Matharu GS, Garriga C, Whitehouse MR, Rangan A, Judge A (2020). Is aspirin as effective as the newer direct oral anticoagulants for venous thromboembolism prophylaxis after total hip and knee arthroplasty? An analysis from the National Joint Registry for England, Wales, Northern Ireland, and the Isle of Man. J Arthroplasty.

[18] Habibi AA, Brash A, Rozell JC, Ganta A, Schwarzkopf R, Arshi A (2024). Aspirin prophylaxis is not associated with increased risk of venous thromboembolism in arthroplasty for femoral neck fractures: a non-inferiority study. Eur J Orthop Surg Traumatol.

[19] Sidhu V, Badge H, Churches T, Maree Naylor J, Adie S, A Harris I (2023). Comparative effectiveness of aspirin for symptomatic venous thromboembolism prophylaxis in patients undergoing total joint arthroplasty, a cohort study. BMC Musculoskelet Disord.

[20] Moore MC, Dubin JA, Bains SS, Hameed D, Nace J, Delanois RE (2024). Trends in deep vein thrombosis prophylaxis after total hip arthroplasty: 2016 to 2021. J Orthop.

[21] Singh G, Prentice HA, Winston BA, Kroger EW (2023). Comparison of 90-day adverse events associated with aspirin and potent anticoagulation use for venous thromboembolism prophylaxis: a cohort study of 72,288 total knee and 35,142 total hip arthroplasty patients. J Arthroplasty.

[22] Sidhu VS, Kelly TL, Pratt N (2022). Effect of aspirin vs enoxaparin on symptomatic venous thromboembolism in patients undergoing hip or knee arthroplasty: the CRISTAL randomized trial. JAMA.

[23] Jacobs JJ, Mont MA, Bozic KJ (2012). American Academy of Orthopaedic Surgeons clinical practice guideline on: preventing venous thromboembolic disease in patients undergoing elective hip and knee arthroplasty. J Bone Joint Surg Am.

